# LX-8000R and UriSed 2 fully automated urine analyzers comparison to manual microscopic examination

**DOI:** 10.5937/jomb0-31711

**Published:** 2022-02-02

**Authors:** Canpolat Erkan Revşa Evin, Özgür Aslan

**Affiliations:** 1 Health Sciences University Diyarbakir Gazi Yaşargil Education and Research Hospital, Department of Medical Biochemistry, Diyarbakir, Turkey

**Keywords:** Automated urine analyzer, LX-8000R, Urised 2, manual microscopy, automatizovani urinski analizator, LX8000R, Urised 2, manuelna mikroskopija

## Abstract

**Background:**

Urinalysis has an important place in evaluating kidney and urinary tract infections. Automated urine analyzers enhance productivity and turnover in laboratories and economize time and labor required for analysis. In the present study, we evaluated and compared analytic and diagnostic performance of UriSed2 with LX-8000R, which is a novel image-based automated urine sediment analyzer.

**Methods:**

A total of 178 urine samples sent to our laboratory were evaluated by the two urine analyzers and standard manual microscopy. Precision and comparison studies were done in accordance with CLSI guidelines.

**Results:**

Sensitivity assessment revealed similar outcomes with both UriSed2 and LX-8000R devices for erythrocyte count (RBC), whereas UriSed2 device yielded higher outcomes for leukocyte count (WBC) and epithelial cells (EPI) than LX-8000R analyzer. Specificity of UriSed2 for WBC and RBC was higher than that of LX-8000R device. According to Gamma statistics, both urine analyzers showed perfect consistency for WBC, RBC and EPI cell counts. Manuel microscopy revealed statistically significant correlation between LX-8000R and UriSed2 in terms of WBC and RBC. Manual evaluation by Bland-Altman analysis demonstrated lower WBC and RBC values and higher EPI as compared to both UriSed2 and LX-8000R devices. As the result of Passing-Bablok regression analysis, both devices were found to be inconsistent with manual microscopy.

**Conclusions:**

We think that evaluation of automated urine analyzers will be more meaningful when they are evaluated together with urine samples and patient clinical findings in addition to comparing with manual microscopy.

## Abstract (sr)

**Uvod:**

Analiza urina ima važno mesto u proceni infekcija bubrega i urinarnog trakta. Automatski analizatori urina povećavaju produktivnost i promet u laboratorijama i štede vreme i rad potreban za analizu. U ovoj studiji smo procenili i uporedili analitičke i dijagnostičke performanse UriSed2 sa LX-8000R, koji je novi automatizovani analizator za analizu sedimenta urina.

**Metode:**

Ukupno 178 uzoraka urina poslanih u našu laboratoriju procenjeno je pomoću dva analizatora urina i standardne ručne mikroskopije. Studije preciznosti i poređenja rađene su u skladu sa smernicama CLSI.

**Rezultati:**

Procena osetljivosti pokazala je slične ishode sa uređajima UriSed2 i LX-8000R za broj eritrocita (RBC), dok je uređaj UriSed2 dao veće rezultate za broj leukocita (WBC) i epitelnih ćelija (EPI) od analizatora LX-8000R. Specifičnost UriSed2 za WBC i RBC bila je veća nego kod LX-8000R uređaja. Prema Gamma statistici, oba analizatora urina pokazala su savršenu konzistenciju za broj ćelija WBC, RBC i EPI. Manuelova mikroskopija otkrila je statistički značajnu korelaciju između LX-8000R i UriSed2 u odnosu na WBC i RBC. Ručna procena Bland-Altmanovom analizom pokazala je niže vrednosti WBC i RBC i veći EPI u poređenju sa UriSed2 i LX-8000R uređajima. Kao rezultat Passing-Bablokove regresijske analize, utvrđeno je da oba uređaja nisu u skladu s ručnom mikroskopijom.

**Zaključak:**

Smatramo da će evaluacija automatizovanih analizatora urina biti značajnija kada se vrednuju zajedno sa uzorcima urina i kliničkim nalazima pacijenata, poredeći ih sa ručnom mikroskopijom.

## Introduction

Urinalysis, which is an important test in clinical medicine, is used for screening, diagnosis and monitoring of urinary system diseases as well as the diseases detected through urinary system [1]. Urinalysis in daily clinical settings is important in terms of detecting hematuria and proteinuria, which are the initial signs of kidney diseases. In addition, it is important also in assessing urinary erythrocyte morphology and in distinguishing a glomerular disease from nonglomerular disease [2]. Urinalysis consists of physical appearance of urine, chemical analysis and microscopic examination of urine sediment. Manual microscopic method for urinalysis is a time-consuming and labor-intensive technique requiring well-trained and experienced technicians [1]. Microscopic examination of urine is affected by a number of factors including speed and duration of centrifugation, amount of urine left in the tube, dye usage, and experience and training of the analyst. Despite all these disadvantages, manual microscopy is the reference method for examination of urine sediment [3]. However, fully automated urine analyzers are recommended by many international institutions for minimizing these effects and for standardization of microscopic analysis [4].

UriSed2 device captures images from the urine centrifuged in a disposable tube by a digital camera attached to a bright-field microscope of 400× magnification. Then the sediment is identified and classified by image-processing software. The automated process used in the UriSed2 analyzer is similar to that used at manual microscopic examination. The images thereafter can be controlled again by a technician, when necessary. UriSed2 software is able to distinguish red blood cells (RBC), white blood cells (WBC), squamous epithelial cells (EPI) and other sediment particles [5] [6].

LX-8000R device works by taking real-time picture of the cells and field image from the urine sample by Flowcell Digital Imaging. It performs the analysis of visible cells in the urine sample and strip analysis simultaneously. It does not require pretreatment or include centrifugation. Because of single-probe distribution system, a small amount of urine is subjected to both microscopic and chemical analyses, which are then reported together. Hence, test time is shortened and working productivity is enhanced providing advantage for pediatric patients as well as patients that have difficulty in giving urine sample [7].

In the literature, no study is encountered about the performance of LX-8000R device. The present study aimed to evaluate analytic performance of the UriSed2 (77 Elektronika Kft, Hungary) and LX-8000R (Hangzhou Longx Technology, Zhejiang, China) automated analyzers by comparing with manual microscopic analysis.

## Materials and Methods

The study was performed using 178 urine samples collected into the clean tubes without preservatives, which have been obtained from the patients applied to the Training and Research Hospital for routine policlinic visit. The urine samples were obtained from the specimens at an amount of 30 mL at the least. The samples were analyzed in small groups on different days. The urine samples, which were divided into three different tubes at an amount of 10 mL for each, were analyzed in an hour at the latest. The study was approved by the Health Sciences Institute Diyarbakır Gazi Yaşargil Training and Research Hospital Ethics Committee (No: 20.12.2019 - 385).

RBC, WBC, EPI, lipid cylinder and crystals (calcium oxalate dihydrate, uric acid, triple phosphate) were studied in both devices and compared with manual microcopy.

### UriSed 2

UriSed2 analyzer pipes the sample from 2 mL urine into a 0.2 mL disposable tube. The tube filled is then centrifuged at 2000 rpm for 10 seconds. Fifteen different fields from various points of the sample can be displayed on the screen by an automated CMOS (Complementary Metal Oxide Semiconductor) fixed cam. The images are recorded in three types as bright-field, phase contrast and composite, and then evaluated by Auto Image Evaluation Module (AIEM). Urinary particles are classified as RBC, WBC, leukocyte clusters, hyaline cylinders, pathologic cylinders, EPI, non-squamous epithelial cells, bacteria, yeast, mucosa, sperm, and sub-crystals (calcium-oxalate monohydrate, calcium-oxalate dehydrate, uric acid or triple phosphate) [8]. Misclassified images can be checked and corrected by the technician, when necessary.

### LX-8000R

It performs the analysis of visible cells in the urine sample and strip analysis at the same time. The samples are put into the device without need for pretreatment. Minimum 3 mL of urine sample is adequate for analysis. The device as well, which does not include centrifugation, is subjected to tube mixing procedure before analysis to provide homogenous mixture of urine. The urine, which is obtained by a single probe at a single step, is subjected to both microscopic and chemical analyses and the results are reported together. For chemical analysis, it is studied by the method of dripping on a strip. Specific Gravity, Color and Turbidity parameters of the urine sample are measured by Refractometry technique. There is a high resolution phase contrast microscope with 10X and 40X lenses on it. Real-time cell picture and images of 20 different fields are taken from the urine by flowcell digital imaging for microscopic analysis. Based on famous light-microscopic morphology detection method, urine tangible ingredient detection combines digital medical image processing technology, computer multimedia technology and artificial intellectualizing technology, which enables detection operators to clearly observe the urine tangible ingredients in the quantitative flow counting chamber under the digital microscope system inserted in Analysis System via large screen display, and then get the detection report of clinical significance according to cells per volume or numbers of tubes. Camera focus settings are automatically made by the software for each sample. Automatic probe wash is performed after each urine sampling (internal and external wash) to prevent contamination of samples. During the procedure, real images of the sample are displayed on the screen without adding any chemical substance into the urine sample. Urinary particles are classified as dysmorphic RBC, RBC, WBC, leukocyte clusters, cylinders, EPI, bacteria, yeast, mucus, sperm and crystals. Other subgroup particles can be defined manually on the screen. In sediment analysis, the parameters are shown on the result screen as XX/µL or p/Hpf [7]. Misclassified images can be checked and corrected by a technician, when necessary.

Technical properties of microscopic and chemical units of the UriSed2 and LX-8000R automated urine analyzers are given in Table 1.

**Table 1:** Technical specifications of the microscopic and the chemical stripe analyzers

	Urised 2	LX-8000R
Microscopic Analyzers		
Throughput	Up to 120 tests/hour	Up to 100 tests/hour
Methodology	Whole view field microscopic image	Flowcell digital imaging
Batch size	100 test tubes	60 test tubes
Min. sample volume	2 mL	3 mL
Detected particle classes	RBC (red blood cells); WBC(white bloodcells and WBC clumps); HYA (hyaline casts); PAT (pathological casts); EPI (squamous epithelial cells); NEC (nonsquamous epithelial cells); BACc (bacteria cocci); BACr (bacteria rods) YEA (yeast) CRY (crystals): [CaOxm (calcium-oxalate monohydrate),CaOxd(calcium-oxalatedihydrate), URI (uric acid), TRI (triple phosphate)]; MUC (mucus); SPRM (sperm); Further classes for manual subclassification are also available!	RBC (red blood cells); Dysmorphic RBC; WBC (white blood cells and WBC clumps); EPI (squamous epithelial cells); BACc (bacteria cocci); YEA (yeast) CRY (crystals); MUC (mucus); SPRM (sperm); Other subgroups particulates can be manually defined on the screen.
Chemical Stripe Analyzers		
Max. throughput	Up to 250 tests/hour	Up to 200 tests/hour
Strips capacity	150 strips	200 strips
Methodology	Reflectance photometer	Reflectance photometer
Min. sample volume	2 mL	3 mL
Memory	Max 10.000 results	Max 100.000 results.
Test wavelengths	4 discrete wavelengths	3 discrete wavelengths
Evaluated parameters	Bilirubin, Urobilinogen, Ketones, Protein, Glucose, Ascorbic acid, Nitrite, Leucocytes, Blood, pH, Specific gravity	Bilirubin, Urobilinogen, Ketones, Protein, Glucose, Ascorbic acid, Nitrite, Leucocytes, Blood, pH, Specific gravity, UMA

### Manual Microscopy

For microscopic examination, 10 mL of urine sample is centrifuged at 2000 rpm (400 g) for 5 min and the sediment obtained was examined within 30 minutes. After eliminating 9 mL of urine sample, the remaining 1 mL urine sample was re-suspended, 20 UL sediment was piped onto a microscope slide and covered with a coverslip (18 × 18 mm). Microscopic examination was done with a light microscope (Olympus, CX21) at 100× and 400× magnifications. The particles were counted per field, and the results were classified semi-quantitatively within intervals or as negative and positive. All urine samples were evaluated by three persons (2 technicians and a medical biochemistry specialist doctor) that have more than 10 years of experience. In order to minimize the inter-observer variability, examinations were performed using the same microscope.

Manual microscopy was used as the reference method for all computations.

### Precision Study

Within-run precision was assessed by measuring RBC and WBC for a total of 20 times in a day using high-level and low-level quality control materials. LX series urine analyzers Urine Dipstick / Microscopics Con trol Level 1-Level 2 (Hangzhou Longxin Technology. Zhejiang, China) was used for LX-8000R device, and KOVA Liqua-Trol with Microscopics Level I-II urinalysis Control (KOVA International. California, United States) was used for UriSed2 device.

Between-run precision was assessed by measuring RBC and WBC for a total of 20 times (5 times in a day for four days) using both quality control materials. Precision of each method was assessed by calculating% variation coefficient (CV%).

### Statistical Analysis

SPSS (Statistical Package for Social Sciences, Chicago, IL, USA) for Windows package program, which is a windows-based software, and MedCalc statistics software (MedCalc Software, Mariakerke, Belgium) were used for the statistical analyses of study data. The data are presented as percentage (%), mean ± standard deviation (SD), correlation coefficient (r), variation coefficient (CV%), 95% confidence interval (95% CI), consistence rate and weighted kappa (κ) (Table 2).

**Table 2:** Semi-quantitative range classification of urine particles

Parameters	Negative	Few	Moderate	High	Many
Erythrocyte<br>(cells/HPF)	0–4	5–10	11–20	21–50	≥51
Leukocyte<br>(cells/HPF)	0–4	5–10	11–20	21–50	≥51
Epithelial cell<br>(cells/HPF)	0–4	5–10	11–20	21–50	≥51

As Urised2 and LX-8000R analyzers use different methods of microscopic analysis, relationship and differences between the results of WBC, RBC and EPI were compared using Passing-Bablok and Bland-Altman graphics. Spearman correlation test was used to investigate the consistency of the microscopic analysis of each device. Correlation coefficient (r) was interpreted as following; <0.3 negligible correlation, 0.3-0.5 low correlation, 0.5-0.7 moderate correlation, 0.7-0.9 high correlation, and >0.9 very high correlation [9]. For gamma statistics and weighted kappa values, it was considered that 0.5-0.75 represents good agreement and >0.75 represents excellent agreement [10]. A p value <0.05 was considered statistically significant.

## Results

The present study compared the data obtained from the analyses of 178 urine samples in two different fully automated urine analyzers.

The sensitivity, specificity, positive predictive values (PPV) and negative predictive values (NPV) of LX-8000R and UriSed 2 as compared to manual urinalysis is demonstrated in Table 3. The sensitivity of UriSed2 device for WBC, RBC and EPI was 90.36%, 63.79% and 70.59%, respectively; and the specificity was 87.23%, 94.12%, and 87.16%, respectively. The sensitivity of LX-8000R device for WBC, RBC and EPI was 84.34%, 65.52% and 51.47%, respectively; and the specificity was 78.72%, 84.87% and 95.41%, respectively.

**Table 3:** Diagnostic accuracy of automated urine analysers compared to manual microscopy

Parameter	Analyzer	Sensitivity	Specificity	PPV (%)	NPV (%)
WBC	LX-8000R	84.34	78.72	77.77	85.05
	UriSed 2	90.36	87.23	86.20	91.11
RBC	LX-8000R	65.52	84.87	67.85	83.47
	UriSed 2	63.79	94.12	84.09	84.21
EPI	LX-8000R	51.47	95.41	87.50	75.91
	UriSed 2	70.59	87.16	77.41	82.60

Within-run and between-run coefficients of RBC and RBC variations for each method are demonstrated in Table 4.

**Table 4:** Within-run and between-run precision of microscopic analysis by LX-8000R and UriSed 2 *SD and CV did not get calculated because the mean value of erythrocyte and leukocyte results was 0

		Within-run precision				Between-run precision			
		Level 1 (Low)		Level 2 (High)		Level 1 (Low)		Level 2 (High)	
Analyzer	Parameter<br>(cells/HPF)	Mean±SD	%CV	Mean±SD	%CV	Mean±SD	%CV	Mean±SD	%CV
LX-8000R	RBC	*	*	33.85±6.20	18.32	*	*	38.45±6.52	16.97
	WBC	*	*	36.10±8.60	23.83	*	*	33.75±9.97	29.55
UriSed 2	RBC	*	*	47.60±7.42	15.59	*	*	38.22±10.18	26.63
	WBC	1.32±0.51	39.29	32.79±4.13	12.60	1.03±0.73	70.58	28.81±4.96	17.22

Gamma statistics comparisons for WBC, RBC and EPI counts between manual urinalysis and LX-8000R and UriSed2 urine analyzers are demonstrated in Table 5 and Table 6. Gamma values for WBC, RBC and EPI were 0.886, 0.782 and 0.770, respectively (p < 0.001) for LX-8000R, and 0.938, 0.880 and 0.805, respectively (p < 0.001) for UriSed2.

**Table 5:** Comparison of the numbers of WBC, RBC and EPI cell counted by the manual method and the LX-8000R

	Number of WBC (cells / HPF)							Number of RBC (cells / HPF)						Number of EPI (cells / HPF)					
Manual Microscopy (cells / HPF)		0–4	5–10	11–20	21–50	≥51	**Total**	0–4	5–10	11–20	21–50	≥51	**Total**	0–4	5–10	11–20	21–50	≥51	**Total**
	0–4	74	18	3	0	0	95	74	18	3	0	0	95	105	3	1	1	0	110
	5–10	6	8	6	0	0	20	6	8	6	0	0	20	11	7	2	0	0	20
	11–20	4	1	5	3	1	14	4	1	5	3	1	14	12	3	2	0	0	17
	21–50	2	1	4	13	3	23	2	1	4	13	3	23	8	9	3	4	0	24
	51	1	0	0	2	23	27	1	0	0	2	23	27	2	3	1	1	0	7
	**Total**	87	28	18	18	27	178	87	28	18	18	27	178	138	25	9	6	0	178
	Gamma	0.886						0.782							0.770				
	p	< 0.001						< 0.001							< 0.001				

**Table 6:** Comparison of the numbers of WBC, RBC and EPI cell counted by the manual method and the UriSed 2

	Number of WBC (cells / HPF)							Number of RBC (cells / HPF)						Number of EP (cells / HPF)					
Manual Microscopy (cells / HPF)		0–4	5–10	11–20	21–50	≥51	**Total**	0–4	5–10	11–20	21–50	≥51	**Total**	0–4	5–10	11–20	21–50	≥51	**Total**
	0–4	102	11	3	3	1	120	113	4	2	1	0	120	96	6	6	1	1	110
	5–10	9	8	0	2	3	22	11	7	2	2	0	22	11	8	1	0	0	20
	11–20	9	1	2	2	0	14	7	2	2	2	1	14	6	5	5	1	0	17
	21–50	0	0	0	1	1	2	0	0	0	1	1	2	3	5	11	5	0	24
	51	2	0	0	1	17	20	3	0	0	1	16	20	0	0	2	2	3	7
	**Total**	122	20	5	9	22	178	134	16	6	7	18	178	116	24	25	9	4	178
	Gamma	0.938						0.880						0.805					
	p	< 0.001						< 0.001						< 0.001					

The correlation between manual urinalysis and LX-8000R and UriSed2 devices was r = 0.731 and 0.793, respectively for WBC (p<0.001); r = 0.554 and 0.639, respectively for RBC (p<0.001), and r = 0.601 and 0.615, respectively for EPI (p<0.001), and these correlations were statistically significant (Table 7).

**Table 7:** The correlation coefficients of microscopic analysis results between the manual microscopy and automated analyzers *r; Spearman correlation of coefficient, p< 0.001

Parameters		LX-8000R<br>r*	UriSed 2<br>r*
Manual<br>Microscopy	RBC	0.554	0.639
	WBC	0.731	0.793
	EPI	0.601	0.615

**Figure 1:**
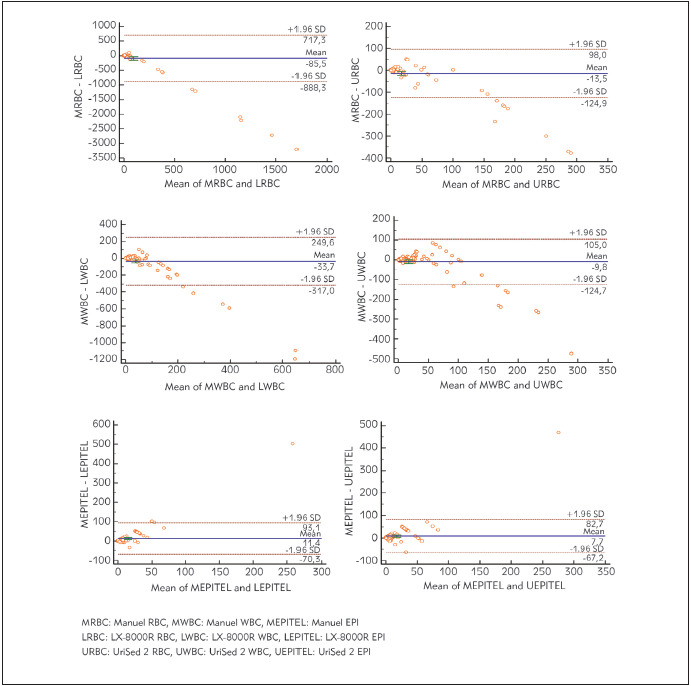
Bland-Altman plots

With Bland-Altman analysis, the mean of differences between manual microscopy and LX-8000R was as following: WBC -33.7, RBC -85.5, and EPI 11.4, whereas the mean of differences between manual microscopy and UriSed2 was as following: WBC -9.8, RBC -13.5, and EPI 7.7. Accordingly, while lower results were obtained for WBC and RBC in both UriSed2 and LX-8000R devices as compared to manual urinalysis, higher results were obtained for EPI (Figure 1).

In the present study, Passing-Bablok regression analysis was used to compare the microscopic analyzers of each device. Regression analysis revealed a linear correlation between manual microscopy and LX-8000R device for WBC (p=0.07). However, it was determined that there is significant deviation from linearity for RBC and EPI (p=0.01 and p=0.01, respectively) with LX-8000R vs. manual microscopy and that manual microscopy and LX-8000R device are not concordance for RBC and EPI count. Results of the regression analysis for the outcomes with manual microscopy vs. UriSed2 device demonstrated significant deviation from linearity for WBC, RBC and EPI (p=0.01, 0.01 and 0.01, respectively), and it was observed that manual microscopy and UriSed2 device are not concordance for WBC, RBC and EPI count (Table 8).

**Table 8:** The results of the Passing-Bablok regression analysis plots for WBC, RBC and EPI counts Comp - comparisons: 1) Manual Microscopy, 2) LX-8000R, 3) UriSed 2

Analytes	Comp	Equation	Intercept (95% CI)	Slope (95% CI)	Cusum<br>p
WBC	1 vs 2	y = 0.00 + 0.60 x	0.00 –(-0.25 to 0.00)	0.60 (0.50 to 0.75)	0.07
	1 vs 3	y = 0.00 + 1.04 x	0.00 –(0.00 to 0.00)	1.04 (-84.92 to 84.92)	0.01
RBC	1 vs 2	y = 0.00 + 0.45 x	0.00 –(0.00 to 0.00)	0.45 (0.23 to 0.58)	0.01
	1 vs 3	y = 0.00 + 0.75 x	0.00 –(0.00 to 0.00)	0.75 (0.52 to 1.00)	0.01
EPI	1 vs 2	y = 0.00 + 5.00 x	0.00 –(0.00 to 0.00)	5.00 (3.40 to 8.50)	0.01
	1 vs 3	y = 0.00 + 2.00 x	0.00 –(0.00 to 0.00)	2.00 (1.45 to 2.58)	0.01

In the present study, uric acid crystal was detected in 4 samples by manual microscopy, whereas it was detected in one sample with LX-8000R device and in none of the samples with UriSed2 device. While manual microscopy detected Ca oxalate crystals in 10 samples, LX-8000R detected Ca oxalate crystals in 9 samples, and UriSed2 device detected in 6 samples. Manuel microscopy detected 1 lipid cylinder, whereas any of the two urine analyzer detected no lipid cylinder.

## Discussion

In urine sediment analysis, automated urine analyzers contribute to the reduction of workforce and potential assay variations and shortening of turnaround time. Manual microscopy remains to be the »gold standard« despite the methodological problems and disadvantages such as that many factors reduce the precision and accuracy of results [11] [12]. Manual microscopy is not only labor-intensive and time consuming, but also has between-technicians differences and low reproducibility [10]. Currently available automated urine analyzers enhance productivity and turnover in the laboratories by increasing the reproducibility and productivity/throughput and are considered to reduce the time and labor required to process urine samples [13] [14].

Bottini et al. [15] stated that UriSed is a precise method with inter- and intra-assay precision ranging from 8%-15%. In addition, they stated that UriSed, depending on the particle count, shows much lower variation than that observed in manual urinalysis. Budak et al. [16] reported higher within-run and between-run CVs with UriSed device as compared to iQ200 and UF-1000 devices. Moreover, they detected slightly higher false-negative RBC and WBC reports with UriSed than the other devices. They suggested sampling and centrifugation steps used in the analytic method of the UriSed device as the origins of this situation. Ma et al. [17] detected that UriSed has acceptable levels of within-run and between-run precision (CV < 20%) for RBC, WBC, Cylinder, EPI and bacteria (BAC). Zaman et al. [6] found within-run precision at low and high control levels to be 17.8% and 6.7%, respectively for RBC and 17% and 4.4%, respectively for WBC. In the present study, the mean value of the LX-8000R and UriSed2 devices' withinrun precision and between-run precision for RBC at low control level was found to be zero; and CV% could not be calculated. Within-run precision at high control level yielded better CV% values for WBC and RBC with UriSed2 device (CV% 12.60 and 15.59, respectively) than LX-8000R device (CV% 23.83 and 18.32, respectively). Between-run precision at high control level yielded better CV% values for WBC with UriSed2 device (CV% 17.22) than LX-8000R device (CV% 29.55), whereas better CV% values with LX-8000R device (CV% 16.97) than UriSed2 device (CV% 26.63) for RBC.

Akin et al. [19] compared the analytic performance of UriSed with that of iQ200 automated analyzer and detected significant correlation between two methods. Laiwejpithaya et al. [1] compared RBC, WBC ve EPI cell counts obtained by UriSed 3 and UX-2000 automated urine analyzers with those obtained by manual microscopy. As a conclusion, they reported that UriSed 3 and UX-2000 devices have almost similar performance for RBC and WBC counts but UriSed 3 is more reliable for EPI cell count [1]. Budak et al. [16] determined consistency by 91.5% between UF-1000i and UriSed, 92.2% between iQ200 and UriSed, and 89.5% between UF-1000i and iQ200 for RBC, whereas they found consistency by 82.2% between UF-1000i and UriSed and 83.7% between iQ200 and UriSed for WBC. In the same study, epithelial cell count demonstrated consistency by 86.6% between UF-1000i and iQ200, 85.1% between UF-1000i and UriSed, 87.6% between iQ200 and UriSed, and 90.2% between UF-1000i and iQ200 [16]. In a study, Ercin [18] compared Urised2 and FUS200 microscopic analyzers and found WBC, RBC and EPI cell counts to be within the same range and reported excellent consistency for these three parameters (Gamma value is 0.916, 0.770 and 0.961, respectively; p <0.001). Jintasuthanont et al. [10] compared the results of UriSed analyzer with that of manual microscopy; they found the gamma value to be 0.837 for WBC, 0.918 for RBC, and 0.939 for squamous epithelial cell count, and they detected high correlation between the results obtained by full-automated analyzer and the results obtained by manual microscopy. In the present study, WBC, RBC and EPI gamma values were 0.886, 0.782 and 0.770, respectively for LX-8000R (p <0.001) and 0.938, 0.880 and 0.805, respectively for UriSed2 (p <0.001). Based on these outcomes, it was determined that both urine analyzers show excellent consistency with manual microscopy for WBC, RBC and EPI cell counts.

Ercin [18] reported high correlation between Urised2-LabUmat2 and FUS200-H800 devices for WBC count, but moderate correlation for RBC count. Moreover, according to the Passing-Bablok regression analysis in this study, they reported that there is no consistency between these two methods because of the presence of remarkable deviation from linearity for WBC and RBC counts (p <0.05 and p=0.01, respectively) but that they are concordance for EPI cell count (p=0.65). In addition, Bland-Altman agreement plot graphics demonstrated that automated microscopy units of the two devices showed acceptable performance for WBC (-29.3 ± 1.96 SD), RBC (43.3 ± 1.96 SD) and EPI (-4.0 ± 1.96 SD) cell counts [18]. Yalçınkaya et al. [3] compared FUS200 and Urised3 urine analyzers where Deming regression analysis yielded a correlation coefficient of 0.961 and 0.961 for WBC and RBC counts, respectively. When they investigated the consistency between two urine analyzers for negative-positive test results, they found the kappa value to be 0.79 (good consistency) for WBC and 0.42 (moderate consistency) for RBC [3]. In the present study, Bland-Altman analysis revealed lower mean of differences between manual microscopy and UriSed2 device for WBC, RBC, EPI (-9.8, -13.5 and 7.7, respectively) suggesting that manual microscopy and UriSed2 device are concordant. The mean of differences between manual microscopy and LX-8000R device for WBC, RBC and EPI was-33.7,-85.5 and 11.4, respectively suggesting that manual microscopy and LX-8000R device are not concordant for WBC and RBC counts but more concordant only for EPI. As a method similar to manual microscopy, centrifuging the urine in the UriSed2 device and screening 15 fields equivalent to 400x magnification and then having the images checked by a technician suggests that the outcomes might be more concordant.

Passing-Bablok regression analysis demonstrated a linear correlation between manual urinalysis and LX-8000R device for WBC (p= 0.07) but remarkable deviation from linearity for RBC and EPI (p=0.01 and p=0.01, respectively). Regarding manual microscopy vs. UriSed2, it was determined that there is a remarkable deviation from linearity for WBC, RBC and EPI cell counts (p=0.01, 0.01 and 0.01, respectively) and that manual microscopy and UriSed2 device are not concordant for WBC, RBC and EPI cell counts. Considering these outcomes, we can say that both devices, in general, are not concordant with manual microscopy; however, this might result from semiquantitative evaluation by manual microscopy.

According to Yalçınkaya et al. [3], urine samples can be analyzed in UriSed 3 and FUS-200 and give reproducible outcomes, UriSed 3 have higher specificity for RBC and higher sensitivity for WBC than FUS-200. They determined that FUS-200 analyzer have higher PPV for WBC but lower PPV for RBC than UriSed 3. They reported almost perfect and similar NPV with FUS-200 and UriSed 3 analyzers for both cell types [3]. Ma et al. [17] detected >80% sensitivity and specificity for RBC, WBC and EPI cells between UriSed device and manual microscopy. Mittal et al. [2] determined the sensitivity, specificity, PPV and NPV of UriSed2 urine analyzer to be 89.2%, 93.3%, 96.8% and 78.8%, respectively for RBC and to be 86%, 91%, 96% and 74.3%, respectively for WBC. In the present study, while UriSed2 device was more sensitive for WBC and EPI (90.36% and 70.59%) than LX-8000R device (84.34% and 51.47%), both devices have nearly similar sensitivity for RBC (63.79% and 65.52%). UriSed2 device had higher specificity, PPV and NPV for WBC and RBC (87.23% and 94.12%; 86.20% and 84.09%; and 91.11% and 84.21%, respectively) than LX-8000R device (78.72% and 84.87%; 77.77% and 67.85%; and 85.05% and 83.47%, respectively).

Bottini et al. [15] stated that UriSed urine analyzer is a precise and accurate alternative to microscopy. They stated also that routine use of these automated urine analyzers would enable better workflow and reduce the turnaround time and, in addition, examination of the images displayed on the screen of UriSed device would potentially eliminate the need for microscopic examination for most of the urine samples [15].

The fact that the LX-8000R device, which is evaluated in the present study, obtains images from 20 different fields in the urine sample by real-time cell picture and field image method without centrifugation can be considered as a limitation. We think that better analysis can be achieved with higher number of images. We also think that evaluation of images from 15 different fields in a centrifuged urine using UriSed2 is a better approach for urinalysis. Despite overall outcomes and statistics, we suggest that evaluation of automated urinalysis devices together with samples and patient clinical findings in addition to comparing with manual microscopy will be more meaningful.

Today, although automated urine analyzers reduce workload in the laboratories, they need to be developed further for they can accurately recognize the pathological elements occurring in the urine due to methods and software.

## Dodatak

### Conflict of interest statement

All the authors declare that they have no conflict of interest in this work.

## References

[1] Laiwejpithaya S, Wongkrajang P, Reesukumal K, Bucha C, Meepanya S, Pattanavin C, et al (2018). UriSed 3 and UX-2000 automated urine sediment analyzers vs manual microscopic method: A comparative performance analysis. J Clin Lab Anal.

[2] Mittal A, Sharma S (2019-May). Comparison of Urised 2 Fully Automated Urine Analyzer to Manual Urine Mıcroscopy. Indıan Journal of Research.

[3] Yalçınkaya E, Erman H, Kıraç E, Şerifoğlu A, Aksoy A, İşman F K, et al (2019). Comparative performance analysis of Urised 3 and DIRUI FUS-200 automated urine sediment analyzers and manual microscopic method. Medeniyet Medical Journal.

[4] Huysal K, Üstünda Y (2015). Otomatik İdrar Analizörleri: Mikroskobik Bakı. Türk Klinik Biyokimya Derg.

[5] Block D R, Lieske J C (2012). Automated Urinalysis in the Clinical Lab. Med Lab Obs (MLO).

[6] Zaman Z, Fogazzi G B, Garigali G, Croci M D, Bayer G, Kránicz T (2010). Urine sediment analysis: Analytical and diagnostic performance of sediMAX®: A new automated microscopy image-based urine sediment analyser. Clin Chim Acta.

[7] 7 http://www.longx.com.cn/en/index.asp.

[8] Mukakab M M (2012). Statistics Corner: A guide to appropriate use of Correlation coefficient in medical research. Malawi Med J.

[9] Jintasuthanont P, Khejonnit V, Opaskiattikul N, Chinswangwatanakul W, Gonggetyai V (2010). Evaluation of the Performance of the Automated Urine Sediment Analyzer 'Urised' Compared with the Manual Method. Siriraj Med J.

[10] Cho J, Oh K J, Jeon B C, Lee S G, Kim J H (2019 Oct 25). Comparison of five automated urine sediment analyzers with manual microscopy for accurate identification of urine sediment. Clin Chem Lab Med.

[11] Carlson D A, Statland B E (1988). Automated Urinalysis. Clin Lab Med.

[12] Wesarachkitti B, Khejonnit V, Pratumvinit B, Reesukumal K, Meepanya S, Pattanavin C, et al (2016). Performance Evaluation and Comparison of the Fully Automated Urinalysis Analyzers UX-2000 and Cobas 6500. Lab Med.

[13] Zaman Z (2015). Automated urine screening devices make urine sediment microscopy in diagnostic laboratories economically viable. Clin Chem Lab Med.

[14] Bottını P V, Martınez M H M, Garlıpp C R (2014). Urinalysis: Comparison between Microscopic Analysis and a New Automated Microscopy Image-Based Urine Sediment Instrument. Clin Lab.

[15] Budak Y U, Huysal K (2011). Comparison of three automated systems for urine chemistry and sediment analysis in routine laboratory practice. Clin Lab.

[16] Ma J, Wang C, Yue J, Li M, Zhang H, Ma X, et al (2013). Clinical Laboratory Urine Analysis: Comparison of the UriSed Automated Microscopic Analyzer and the Manual Microscopy. Clin Lab.

[17] Ercin U (2020). A Comparative study on the performances of 77 Elektronika Urised 2-LabUmat 2 and Dirui FUS 200-H 800 urine analyzers. International Journal of Medical Biochemistry.

[18] Akin O K, Serdar M A, Cizmeci Z, Genc O, Aydin S (2009). Comparison of LabUMat-with-UriSed and iQ®200 fully automatic urine sediment analysers with manual urine analysis. Biotechnol Appl Biochem.

[NaN] 8 UriSed 2 Fully Automated Urine Sediment AnalyzerUsermanual for SW version 2.0.4. 77 Elektronika Kft.

